# Detection of copy number variations based on a local distance using next-generation sequencing data

**DOI:** 10.3389/fgene.2023.1147761

**Published:** 2023-09-22

**Authors:** Guojun Liu, Hongzhi Yang, Zongzhen He

**Affiliations:** ^1^ School of Mathematics, Xi’an University of Finance and Economics, Xi’an, China; ^2^ Department of Radiology, XD Group Hospital, Xi’an, China

**Keywords:** copy number variation, next-generation sequencing, read depth, local distance, normal distribution

## Abstract

As one of the main types of structural variation in the human genome, copy number variation (CNV) plays an important role in the occurrence and development of human cancers. Next-generation sequencing (NGS) technology can provide base-level resolution, which provides favorable conditions for the accurate detection of CNVs. However, it is still a very challenging task to accurately detect CNVs from cancer samples with different purity and low sequencing coverage. Local distance-based CNV detection (LDCNV), an innovative computational approach to predict CNVs using NGS data, is proposed in this work. LDCNV calculates the average distance between each read depth (RD) and its *k* nearest neighbors (KNNs) to define the distance of KNNs of each RD, and the average distance between the KNNs for each RD to define their internal distance. Based on the above definitions, a local distance score is constructed using the ratio between the distance of KNNs and the internal distance of KNNs for each RD. The local distance scores are used to fit a normal distribution to evaluate the significance level of each RDS, and then use the hypothesis test method to predict the CNVs. The performance of the proposed method is verified with simulated and real data and compared with several popular methods. The experimental results show that the proposed method is superior to various other techniques. Therefore, the proposed method can be helpful for cancer diagnosis and targeted drug development.

## 1 Introduction

Copy number variation (CNV) is one of the important types of structural variation (SV) in the human genome, which enables humans to induce cancer and complex diseases (Mccarroll and Altshuler, 2007; Beroukhim et al., 2010; Pos et al., 2021). Compared to other types of SVs, CNV belongs to a medium-to-large-scale SV, which can lead to the deletion or amplification of genomic fragments that are not less than 1 kb in length compared to the reference genome (Freeman et al., 2006; Yuan et al., 2018). The human genome has a high rate of CNVs (Redon et al., 2006; Handsaker et al., 2015), which directly leads to changes in the base content and influences the expression level of genes and structural reorganization of the human genome (Sebat et al., 2004; Sharp et al., 2005; Nowakowska, 2017). Therefore, accurate detection of CNVs is crucial for clinical diagnosis, localization of oncogenes, and targeted drug development. The emergence of next-generation sequencing (NGS) technology has made it possible to realize precise CNV detection. Compared with conventional detection technology (array comparative genomic hybridization and fluorescence *in situ* hybridization) (Carter, 2007; Buysse et al., 2009), NGS technology has many advantages such as high speed, low cost, high-throughput, flexible sequencing coverage, and high resolution (Shendure and Ji, 2008; Meyerson et al., 2010). Due to the complexity of the structure of CNVs and the alignment errors, the existing methods still have low accuracy in detecting CNVs from cancer samples with different purity and low sequencing coverage.

To date, the vast majority of CNV detection methods are developed based on read depth (RD) strategy. The basic principle of RD strategy is that the number of short reads aligned to the reference genome is proportional to the number of copies at that position (Yoon et al., 2009). The advantage of this strategy is that it can in principle predict copy number gain and loss of any size, but its drawback is the low resolution of breakpoint detection. A large number of CNV detection methods have been developed based on RD strategy, including CNV-LOF (Yuan et al., 2021), SeqCNV (Chen et al., 2017), BIC-seq2 (Xi et al., 2016), dpCNV (Xie et al., 2021), iCopyDav (Dharanipragada et al., 2018), CNVnator (Abyzov et al., 2011), SPCNV (Liu et al., 2023), CNVkit (Talevich et al., 2016), among others. CNV-LOF performs successive and non-overlapping divisions of RD profiles to form a set of RD segments, and performs the cyclic binary segmentation (CBS) algorithm (Venkatraman and Olshen, 2007) on each segment. CNV-LOF uses local outlier factor algorithm to assign an abnormal score for each RD segment, and declares CNVs using a boxplot procedure. Its performance in detecting low-coverage samples is uneven between recall and precision, and it is insensitive to detecting low purity cancer samples. SeqCNV extracts RDs from paired samples, constructs a maximum penalized likelihood estimation model, and chooses an appropriate threshold to predict CNVs. It is sensitive to detecting copy number gains and not suitable for detecting copy number losses. BIC-seq2 can preprocess sequencing data at the nucleotide level and uses bayesian information criteria (BIC) to segment RD profiles and predict CNVs. When it detects low purity cancer samples, its performance is balanced between recall and precision. However, when detecting medium and high purity cancer samples, recall far outperforms precision. dpCNV uses the density peak clustering algorithm to extract two features of RD, namely, the minimum distance and local density, then uses these features to fit a gaussian distribution and estimate a probability value for each RD segment to finally predict CNVs using hypothesis testing. Due to the bias between the distribution of features and distribution of fitting, dpCNV detects a large number of CNVs but with low precision, especially in the detection of high-purity cancer samples. iCopyDav can automatically evaluate or customize bin size, perform GC content and mappability bias calibration on RD profiles, and use the CBS algorithm to segment preprocessed RD profiles and predict CNVs. iCopyDav is not suitable for the detection of low-purity cancer samples. CNVnator performs GC content calibration for each bin, and uses the mean-shift approach with multiple bandwidths to segment RD files and predict CNVs. CNVnator is suitable for the detection of long CNVs and high-purity cancer samples, and not sensitive to breakpoint detection, which results in a large number of false positives in the test results. SPCNV uses the k nearest neighbors of each RD to define the shortest path, shortest path relationship and shortest path weight. Based on the above definitions, SPCNV further constructs the relative shortest path score. Each RD is assigned a relative shortest path score, and the boxplot is used to predict CNVs. It is insensitive to detect short CNVs in low-purity cancer samples. CNVkit extracts the targeted reads and the non-specially captured off-target reads to predict CNVs. Its performance is unbalanced between recall and precision, and it is not suitable for detecting low-purity cancer samples and copy-number deletion areas. Based on the analysis and discussion of the aforementioned methods, the performance of the methods shows low robustness when detecting cancer samples with different purity and low coverage.

In this work, a new method for CNV detection called LDCNV (local distance-based CNV detection method) is proposed along with consideration of the problems with existing methods. It is capable of efficiently identifying CNVs across the whole genome using NGS data. The core idea of LDCNV is constructed based on the idea of local distance (Zhang et al., 2009). First, LDCNV uses the mean of the distances between each RD and its k nearest neighbors to define a KNN distance, and the mean of the distances between the k nearest neighbors to define a KNN internal distance. Second, a local distance score (LDS) is constructed using the ratio between the KNN distance and KNN internal distance. Finally, each RD is assigned an LDS. Based on the LDS profile, we use the normal distribution to evaluate the *p*-value of each RD, and the hypothesis test to predict CNVs (Yuan et al., 2021). Compared with existing techniques, the main contributions of the proposed method are as follows: 1) Due to the comparison error, GC content deviation, sequencing error, repetitive regions, sample contamination and low sequencing coverage, the difference between normal RDs and abnormal RDs signals is not obvious, which will lead to some locally insignificant CNVs being considered normal events. The local distance is more conducive to the recognition of the CNVs than traditional methods. 2) By fully considering the global RD trend and the local difference between each RD and its neighboring RDs, we calculate the average distance between an RD and its k nearest neighbors, and the average distance between the k nearest neighbors. Based on the above calculation, we further construct the LDS, which objectively reflects the abnormality of each RD. The degree of divergence between an RD and its k nearest neighbors is observed from a local perspective, which is more advantageous to discover CNVs than traditional methods.

The remainder of this article is organized as follows. Section 2 includes the workflow of LDCNV, data preprocessing, construction of LDS, and output of CNVs. In section 3, simulation and real data are used to verify the performance of the proposed method, various well-known methods are selected for comparison, and the experimental results are analyzed and discussed. Section 4 summarizes this work, points out the shortcomings of the proposed method, and discusses future work perspectives.

## 2 Method and materials

### 2.1 Workflow of LDCNV

**FIGURE 1:**
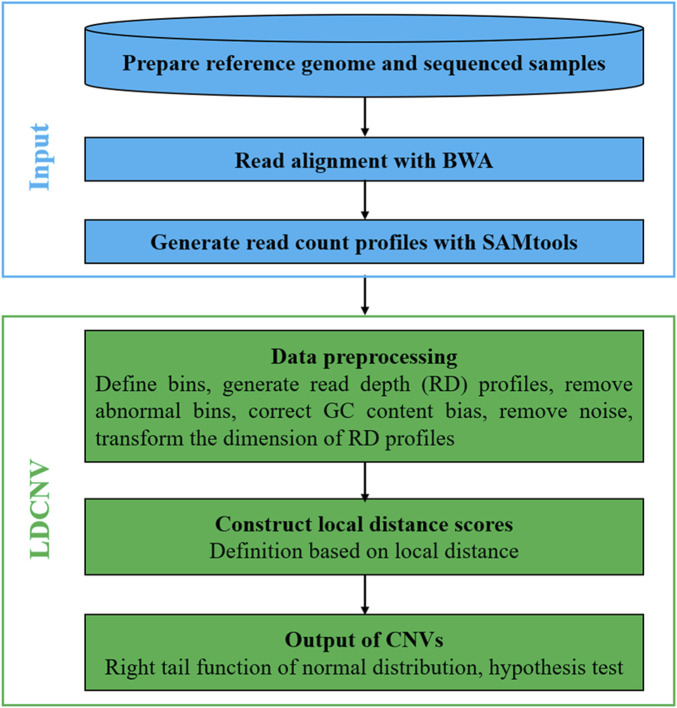
The workflow of LDCNV.

LDCNV is an RD-based CNV detection method that can efficiently identify CNVs from whole-genome sequencing data. Figure 1 describes the LDCNV workflow in details. The sequenced sample is a Fastq format file, which consists of a large number of short reads. The short reads are aligned to the reference genome using the BWA tool (Li and Durbin, 2010), which generates sequence alignment files. The read count (RC) profiles are extracted from sequence alignment files using the SAMtools tool (Li et al., 2009). A bin program is executed on the RC profiles, which generates the RD profiles. The preprocessing of RD profiles mainly includes filtering bins containing “N" positions, calibrating the GC content bias, removing noise, and transforming the dimensions of RD profiles. Based on the preprocessed RD profiles, LDS is constructed based on the theory of local distance, and LDCNV allocates an LDS for each RD. Based on the LDS profiles, the right tail function of the normal distribution is used to evaluate the *p*-value of each RD, and then we use the hypothesis test to predict CNVs. In addition, LDCNV is developed using Python and R languages, and its source code can be downloaded at https://github.com/gj-123/LDCNV/releases.

### 2.2 Data preprocessing

The main purpose of the preprocessing step is to obtain a reasonable and valid RD profile. First, the BWA tool is used to align the sequencing reads to the reference genome in order to generate a sequence alignment file. The RC profile is extracted from the sequence alignment file using the SAMtools tool and expressed using Eq. 1.

$$RC=\left\{RC_{1},RC_{2},RC_{3},\cdot \cdot \cdot ,RC_{n}\right\},$$ (1)

where $RC_{n}$ represents the value of the n-th RC, which is equal to the number of reads on the n-th position of the reference genome. Next, a bin program (Yuan et al., 2021) is executed on the RC profile, which is divided into consecutive and non-overlapping partitions to generate an RD profile. This process is described in Eq. 2.

$$RD=\left\{RD_{1},RD_{2},RD_{3},\cdot \cdot \cdot ,RD_{j}\right\},$$ (2)

where $RD_{j}$ represents the value of the j-th RD, which is equal to the mean RC in the j-th bin. The reference genome contains a large number of “N" positions that represent uncertain base positions during the sequencing process. The reads cannot be aligned to the positions, which will lead to the misinterpretation that copy number loss occurs at the positions. Here, the strategy of published methods is adopted to filter out the bins that contain “N" positions (Yuan et al., 2021). At the same time, the median method (Yoon et al., 2009) is used to calibrate the GC content in each bin, which is described by Eq. 3.

$$RD_{i}^{\prime }=\frac{RD_{i}\cdot RD_{sum}^{m}}{RD_{gc}^{m}},$$ (3)

where $RD_{i}^{\prime }$ represents the calibrated value of the i-th RD, $RD_{sum}^{m}$ denotes the mean of all RDs, $RD_{i}$ represents the value of the i-th original RD, and $RD_{gc}^{m}$ denotes the mean of RDs that possess the same GC content. The total variation model (Condat, 2013; Duan et al., 2013) denoises the RD profile to generate an RD segment (RDS) profile. The one-dimensional RDS profile is converted into a two-dimensional $RDS^{\prime }$ profile, which consists of RDS ratios and differences between adjacent RDS ratios and is described by Eq. 4 (Liu et al., 2020).

$$RDS^{\prime }=\left\{\left(RDSH_{i},RDSL_{i}\right)|i\in N^{*},1\leq i\leq n\right\},$$ (4)

where $RDSH_{i}$ represents the i-th RDS ratio, $RDSL_{i}$ denotes the i-th differences between adjacent RDS ratios. The first dimension can observe the magnitude of RDs from a global perspective, and the second dimension can observe the local difference of RDs from a local perspective. The two dimensions are very beneficial to find insignificant CNVs, and provide an effective dataset for the next step to build a local distance score.

### 2.3 Construction of local distance scores

With the RDS’ profile, an LDS is computed for each RDS. Here, each element in the RDS’ is treated as an object x. The LDS reflects the degree of isolation of an object relative to its k nearest neighbors from a local perspective. The principle of LDS is very suitable for detecting CNVs especially some locally insignificant ones. The LDS of an object depends on the ratio of the mean of the distances between the object and its k nearest neighbors to the mean of the distances between its k nearest neighbors. Before constructing an LDS, it is necessary to introduce some related theories and definitions (Zhang et al., 2009), which mainly include the *k*-distance, the *k*-distance neighborhood, KNN distance, and KNN internal distance of an object.

Definition 1 (*k*-distance of x): The *k*-distance of an object x is defined as

$$k-dist\left(x\right)=dist\left(x,y\right),$$ (5)

where x represents any object in RDS’, y represents the k-th object closest to x in ${\text{RDS}}^{\prime }\backslash \left\{x\right\}$ in ascending order of distance, $dist\left(x,y\right)$ represents the Euclidean distance between x and y, *k* represents a positive integer.

Definition 2 (*k*-distance neighborhood of x): The k-distance neighborhood of an object x is defined as a set of objects, and the distance defined in Eq. 6 between each object in the set and x is no greater than $k-dist\left(x\right)$ .

$$S_{k-dist}\left(x\right)=\left\{z|z\in {\text{RDS}}^{\prime }\backslash \left\{x\right\},dist\left(x,z\right)\leq k-dist\left(x\right)\right\}.$$ (6)

Definition 3 (KNN distance of x): The KNN distance of an object x is defined in Eq. 7 as the average distance from x to those objects in $S_{k-dist}\left(x\right)$ .

$$knn-dist\left(x\right)=\frac{1}{k}\sum_{z_{i}\in S_{k-dist}\left(x\right)}dist\left(x,z_{i}\right),$$ (7)

where x represents any object in ${\text{RDS}}^{\prime }$ , and $z_{i}$ represents the i-th object in $S_{k-dist}\left(x\right)$ .

Definition 4 (KNN internal distance of x): The KNN internal distance of an object x is defined in Eq. 8 as the average distance between objects in $S_{k-dist}\left(x\right)$ .

$$knn-indist\left(x\right)=\frac{1}{k\left(k-1\right)}\sum_{z_{i},z_{j}\in S_{k-dist}\left(x\right),i\neq j}dist\left(z_{i},z_{j}\right),$$ (8)

where $z_{i}$ and $z_{j}$ represent the i-th and j-th objects in $S_{k-dist}\left(x\right)$ , respectively.

Definition 5 (LDS of x): The LDS of an object x is defined in Eq. 9 as the ratio between $knn-dist\left(x\right)$ and $knn-indist\left(x\right)$ .

$$LDS\left(x\right)=\frac{knn-dist\left(x\right)}{knn-indist\left(x\right)},$$ (9)

where $LDS\left(x\right)$ represents LDS of x, which is defined as a ratio between KNN distance and KNN internal distance of x. The larger the value of LDS, the more likely the object is a CNV, which indicates that the object is isolated from its k nearest neighbors. The smaller the value of LDS, the more likely the object is a normal one, which means that the object is close to its k nearest neighbors.

**FIGURE 2:**
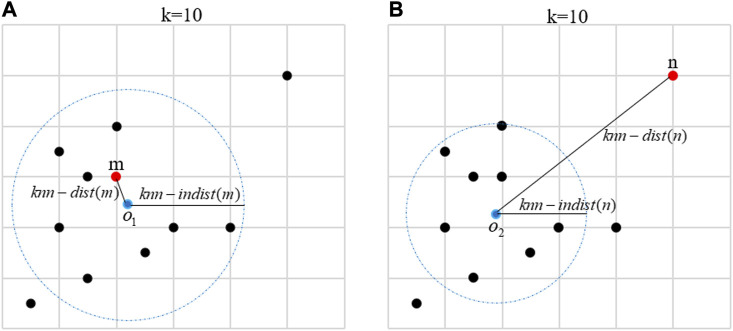
**(A)** The construction process of the local distance score of a normal object *m*. The red point represents a normal object *m*, all black points represent the 10 nearest neighbors of *m*, the blue point $o_{1}$ represents the center of all nearest neighbors, and the blue dotted circle represents the reformed nearest neighbor region. The distance between *m* and $o_{1}$ represents $knn-dist\left(m\right)$ , and the radius of the blue dotted circle represents $knn-indist\left(m\right)$ . **(B)** The construction of a local distance score for an abnormal object *n*. The red point represents an abnormal object *n*, all black points represent the 10 nearest neighbors of *n*, the blue point $o_{2}$ represents the center of all nearest neighbors, and the blue dashed circle represents the reformed nearest neighbor region. The distance between *n* and $o_{2}$ represents $knn-dist\left(n\right)$ , and the radius of the blue dotted circle represents $knn-indist\left(n\right)$ .

To further illustrate the core idea of LDS, the following example describes the KNN distance, KNN internal distance, and LDS of a normal object and an abnormal object in details. As shown in Figure 2A, the red points represent a normal object *m*, all black points represent the 10 nearest neighbors of *m*, and the blue dot $o_{1}$ represents the center position of all black points. $knn-dist\left(m\right)$ is equal to the distance between *m* and $o_{1}$ . The blue dashed circle with $o_{1}$ as the center represents the reconstructed nearest neighbor region of *m*, and $knn-indist\left(m\right)$ is equal to the radius. From Figure 2A, it can be seen that *m* is close to most of its nearest neighbors, and $knn-dist\left(m\right)$ is much smaller than $knn-indist\left(m\right)$ . Therefore, since the LDS of *m* is very small, it can be inferred that *m* is a normal area. In Figure 2B, the red points represent an abnormal object *n*, all black points represent the 10 nearest neighbors of *n*, and blue dot $o_{2}$ represents the center position of all black points. $knn-dist\left(n\right)$ is equal to the distance between *n* and $o_{2}$ . The blue dashed circle with $o_{2}$ as the center represents the reconstructed nearest neighbor region of *n*, and $knn-indist\left(n\right)$ is equal to the radius. From Figure 2B, it can be seen that *n* is isolated by its nearest neighbors, and $knn-dist\left(n\right)$ is much larger than $knn-indist\left(n\right)$ . Therefore, since the LDS of *n* is very large, it can be inferred that *n* is a CNV.

### 2.4 Inference of CNVs

After calculating the LDS for each object, it cannot be determined whether the objects are CNVs or normal ones yet. Therefore, we need to choose a reasonable cutoff strategy to distinguish between normal and abnormal objects. In this step, we evaluate the significance level for each LDS. First, we define a null hypothesis that there is no CNVs in the genome, and then all LDSs fit a normal distribution $LDS∼N\left(\mu_{LDS},\sigma_{LDS}^{2}\right)$ . Secondly, we calculate the *p*-value for each LDS using the right tail function of the normal distribution, which is described by using Eq. 10.

$$P\left(x\right)=\frac{1}{\sqrt{2\pi }\sigma_{LDS}}\int_{x}^{+\infty }e^{-\frac{{\left(t-\mu_{LDS}\right)}^{2}}{2{\sigma_{LDS}}^{2}}}dt,$$ (10)

where $\mu_{LDS}$ represents the mean value of LDSs, and $\sigma_{LDS}^{2}$ represents the variance of LDSs. Here, we choose the right tail function of the normal distribution to calculate the *p*-value of each LDS. The higher the LDS, the more likely the object is to be a CNV. Finally, we choose a significance level α as the baseline for judging CNVs. If the LDS of an object is greater than α, it is considered as a CNV. Along with the declared CNVs, the type of CNVs (gain and loss) is distinguished. Here, the mean value of RD of normal objects is taken as the baseline for judging gain and loss. If the RD of an abnormal object is greater than the mean RD of normal objects, it is considered as a gain. Otherwise, it is considered as a loss.

## 3 Results and discussion

With the construction of LDCNV, it is very important to design a reasonable and efficient experimental scheme to verify the performance of the proposed method. In this work, simulation and real data are used to test the performance of the proposed method, and five well-known methods (SPCNV, CNV-LOF, CNVnator, BIC-seq2, and CNVkit) are used for comparison. In the simulation data experiments, we evaluate the performance of each method from three perspectives. First, four datasets with different configurations are used to analyze and discuss the performance of each method in terms of recall, precision, and F1-score. Secondly, we analyzed and discussed the true positive rate (TPR) and false discovery rate (FDR) of each method to detect CNVs of different sizes. Finally, we analyze the influence of the selection of k parameter on the performance of the proposed method. In the real data experiments, six real human data samples are selected from 1000 Genomes Project to test the performance of the proposed method and the five comparison methods. The detection results of previous studies of the samples are recorded in the Database of Genomic Variants (DGV) (Macdonald et al., 2014), which can be used as ground truth to approximate the calculation of three performance metrics (recall, precision, and F1-score) for each method. In order to verify the operation efficiency of the proposed method, LDCNV and other comparison methods are executed on a group of samples, and the operation time of each method is analyzed and discussed at the same time.

### 3.1 Simulation data experiments

A comprehensive genome structural variation simulation software called IntSIM (Yuan et al., 2017) is employed to generate multiple sets of simulation datasets with different configurations. Each simulated sample has two key parameters: tumor purity (TP) and sequencing coverage (SC). In this study, SC is set to 5x, and TP is set to 0.2, 0.4, 0.6 and 0.8, respectively. Each simulation sample contains a total of 14 CNVs, which are composed of 6 gains and 8 losses, and their lengths range from 10k bp to 50k bp. Under these configuration conditions, a total of 4 sets of simulation data are generated by the software, and each set of data contains 50 samples. To ensure the stability of the test results, three performance indicators of each set of data are averaged over the 50 samples of the dataset.

**FIGURE 3:**
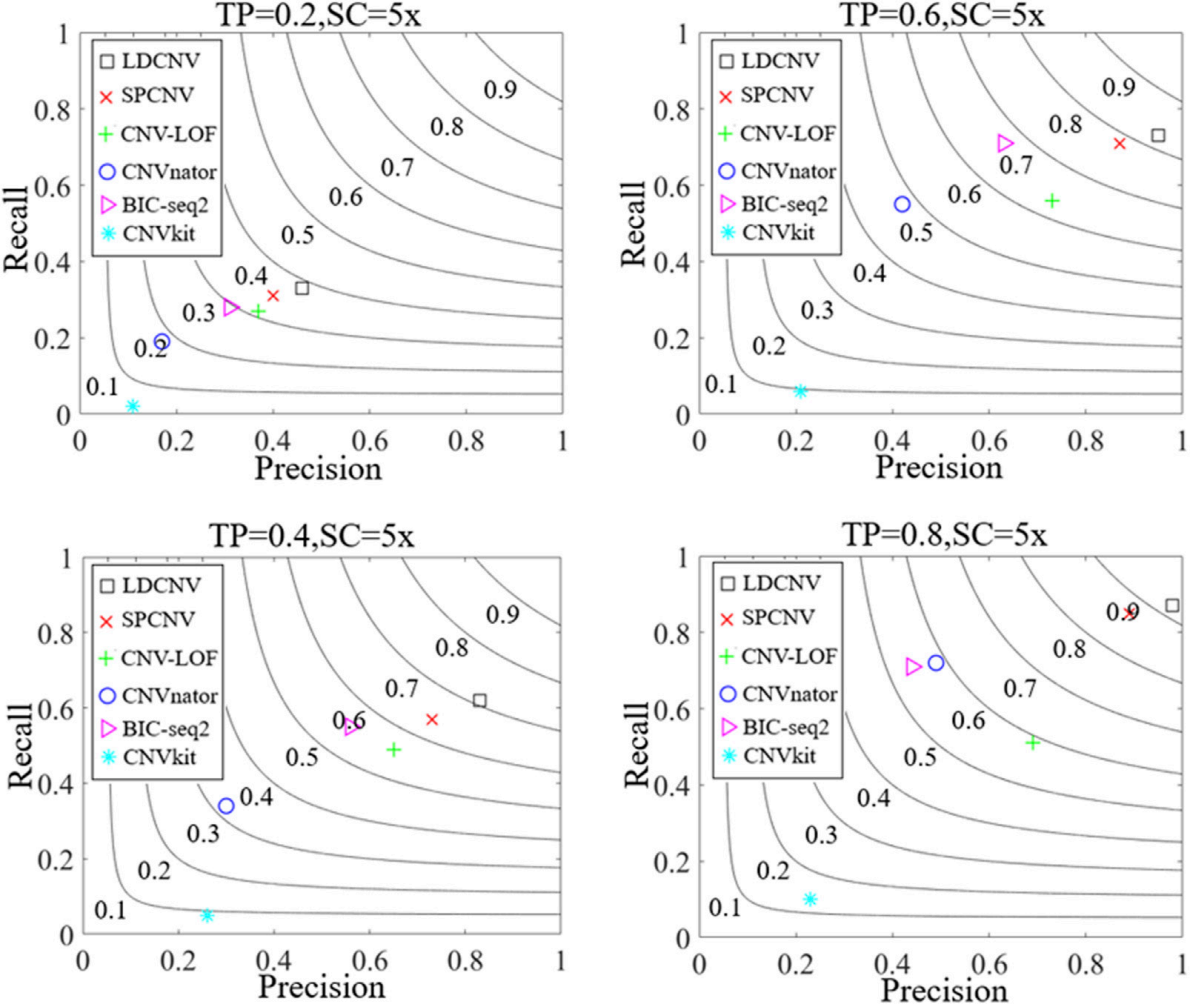
Comparison of LDCNV and five comparison methods in terms of recall, precision, and F1-score, four sets of simulation samples. The F1-score levels are depicted with black arcs from 0.1 to 0.9. The equations on the left and right sides of the comma represent the tumor purity (TP) and sequencing coverage (SC), respectively.

Along with the simulation dataset, the performance of the proposed method and five compared methods is evaluated by calculating recall, precision and F1-score. Recall is defined as the ratio between the number of correctly detected CNVs and the number of simulated CNVs (Magi et al., 2013), precision is defined as the ratio between the number of correctly detected CNVs and the total number of detected CNVs (Magi et al., 2013), and F1-score is defined as the harmonic mean of recall and precision. Figure 3 presents the detection results of each method in details. Overall, the performance of each method shows an upward trend as TP increases gradually. For example, when the TP is equal to 0.2, the recall, precision and F1-score of SPCNV are equal to 0.31, 0.4 and 0.35, respectively. When the TP is equal to 0.8, the recall, precision and F1-score of SPCNV are equal to 0.85, 0.89 and 0.87, respectively. The three performance indicators of SPCNV are increased by about 50%, which indicates that the performance of the method is sensitive to the change of TP. CNVkit get the lowest recall and precision in each group of samples, because it detects CNVs most of which belong to losses. The performance of BIC-seq2 is balanced between recall and precision at low and medium TP. When the TP is equal to 0.8, its recall is far better than precision, and its F1-score is lower than that when TP is equal to 0.6. This shows that BIC-seq2 is not good at detecting high-purity cancer samples. The recall of CNVnator is better than the precision in each group of samples because it detects a large number of long CNVs, most of which are false positive positions. Therefore, CNVnator gets low precision. CNV-LOF gets a medium F1-score when TP is equal to 0.2. F1-scores of CNV-LOF have little change when TP is equal to 0.4, 0.6 and 0.8, respectively. SPCNV obtains better F1-scores than other four comparison methods (CNV-LOF, CNVnator, BIC-seq2, and CNVkit) in each group of samples. In terms of precision, LDCNV gets the best precision in each group of samples, followed by SPCNV, CNV-LOF, and other three methods. In terms of recall, LDCNV obtains the best recall in each group of samples, followed by SPCNV, and other four methods. In general, LDCNV gets the best F1-score in each group of samples, followed by SPCNV, and other four methods.

**FIGURE 4:**
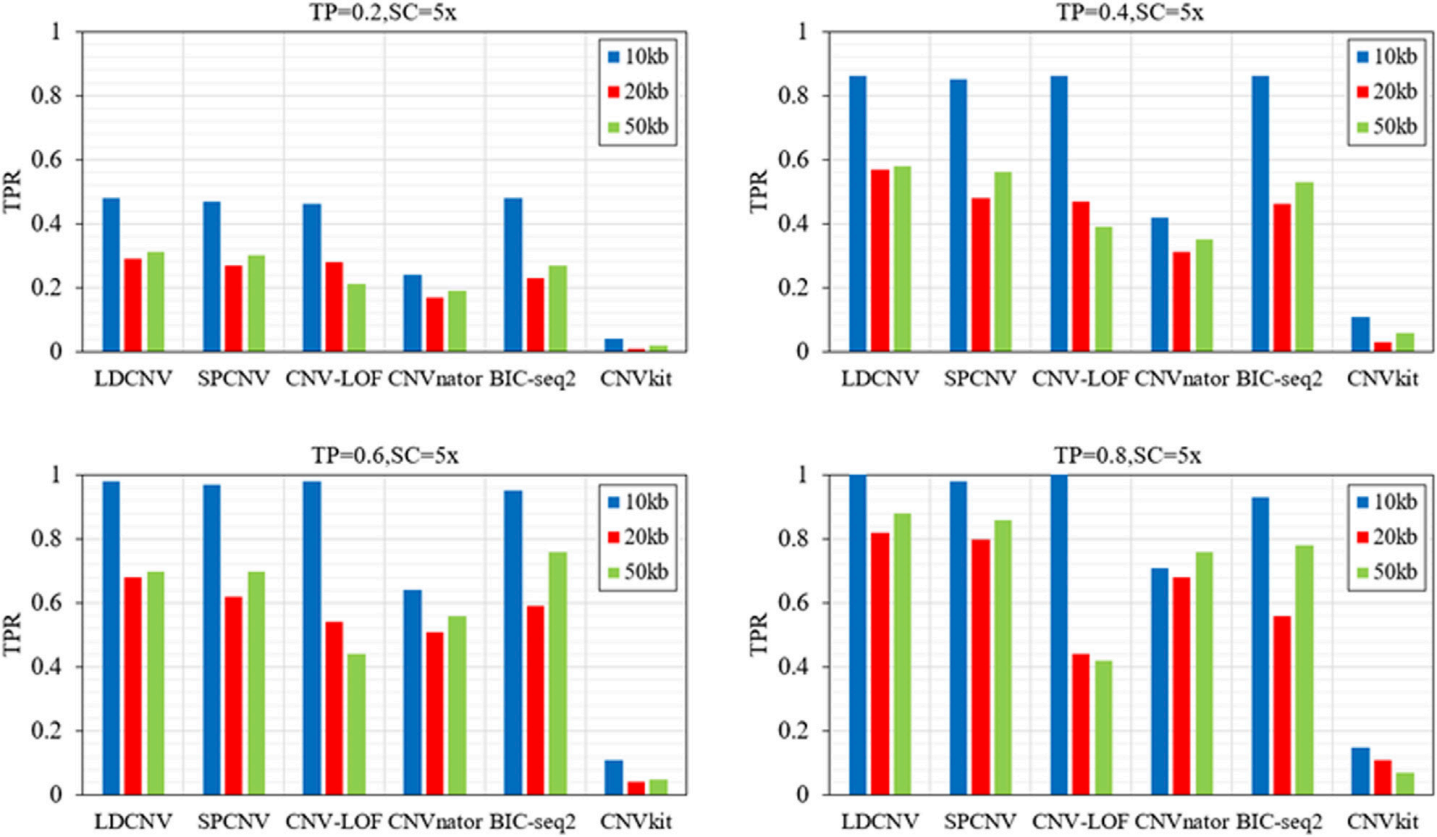
Comparison of TPR of LDCNV and five comparison methods at CNVs of different sizes on four sets of simulation samples. TP and SC represent tumor purity and sequencing coverage, respectively.

**FIGURE 5:**
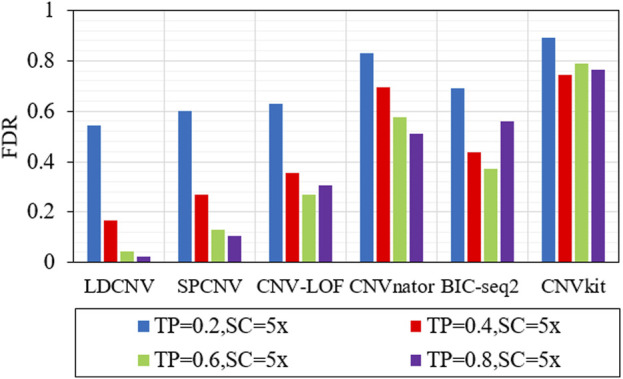
Comparison of FDR of LDCNV and five comparison methods at CNVs of different sizes on four sets of simulation samples. TP and SC represent tumor purity and sequencing coverage, respectively.

In order to verify the performance of each method in detecting CNVs of different sizes (10kb, 20kb and 50 kb), six methods are executed on four groups of samples with different configurations. Here, we use the true positive rate (TPR) and false discovery rate (FDR) to evaluate the performance of each method, and the corresponding comparison results are described in detail in Figure 4 and Figure 5. TPR is equal to the number of correctly detected CNVs divided by the number of all correct CNVs in each length of CNVs. FDR is equal to the number of false positive events divided by the number of all predicted events. Except when TP is equal to 0.6 and CNV size is equal to 50kb, LDCNV gets the best TPR and FDR, respectively. This shows that the sensitivity of LDCNV is the best among the six methods almost in each group of samples. CNVkit obtains the lowest TPR and highest FDR in each group of samples. When TP is equal to 0.2 and CNV size is sequentially equal to 10k, 20k and 50k, TPR of SPCNV ranks third, third and second, respectively. The above analysis shows that the method is suitable for detecting long CNVs. Under the same configuration conditions, TPR of CNV-LOF ranks third, second and fourth, respectively. This implies that CNV-LOF is suitable for detecting medium length CNVs. Similarly, TPR of BIC-seq2 ranks first, fourth and third, respectively. This shows that BIC-seq2 is good at detecting short CNVs. When TP is equal to 0.2, TPR of CNVnator ranks fifth. The performance of CNVnator and CNVkit is lower than other four comparison methods, and the reason for this result is consistent with the above experimental analysis. Except when TP is equal to 0.2, SPCNV‘s TPR ranks second. CNV-LOF and BIC-seq2 get the best TPR at the same time when TP is equal to 0.4 and CNV size is equal to 10k, but their FDRs are lower than LDCNV. when TP is equal to 0.4 and CNV size is sequentially equal to 20k and 50k, TPR of CNV-LOF ranks third and fourth, and TPR of BIC-seq2 ranks fourth and third, respectively. The performance of TPR of CNVnator is consistent with that under the condition that TP is equal to 0.2. When TP is equal to 0.6 and CNV size is equal to 50k, TPR of LDCNV ranks second. When TP is equal to 0.6, TPR of CNV-LOF ranks first, fourth and fourth, and TPR of BIC-seq2 ranks fourth, third and first, respectively. The ranking of TPR of CNVnator is consistent with that under the condition that TP is equal to 0.2. When TP is equal to 0.8 and CNV size is sequentially equal to 10k, 20k and 50k, TPR of CNV-LOF ranks first, fifth and fifth, TPR of CNVnator ranks fourth, third and fourth, and TPR of BIC-seq2 ranks third, fourth and third, respectively. In terms of FDR, LDCNV gets the best FDR, followed by SPCNV, CNV-LOF, and other three methods. In general, the performance of LDCNV in detecting different size CNVs is robust in cancer samples of different purity.

**FIGURE 6:**
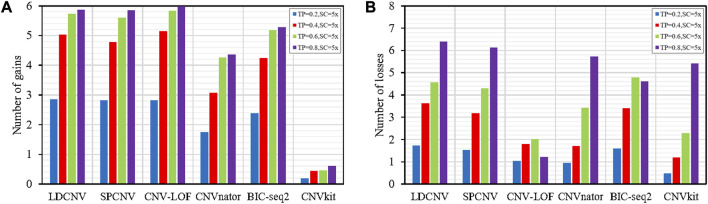
**(A)** Comparison of the number of gains detected by each method. **(B)** Comparison of the number of losses detected by each method.

To verify the performance of each method in detecting gains and losses, we used the number of gains and losses detected by each method as an evaluation indicator. Figures 6A, B provide detailed descriptions of each method for detecting gains and losses, respectively. Except for CNVkit, the remaining methods detect more gains than losses in the vast majority of samples. As the TP increases, the number of gains and losses detected by each method shows an upward trend, indicating that the performance of each method is sensitive to TP. In Figure 6A, we can see that LDCNV detects the most gains with a purity of 0.2. In other cases, LDCNV ranks second. CNV-LOF detects the most CNVs at TPs of 0.4, 0.6, and 0.8, respectively. When TP is equal to 0.2, both CNV-LOF and SPCNV rank second. When TP is sequentially equal to 0.4, 0.6, and 0.8, SPCNV ranks third. BIC-seq2, CNVnator and CNVkit rank fourth, fifth, and sixth, respectively. In Figure 6B, we can see that LDCNV detects the most losses at TPs of 0.2, 0.4, and 0.8, respectively. In other cases, LDCNV ranks second. When TP is equal to 0.6, BIC-seq2 detects the most CNVs. BIC-seq2 ranks second at TP of 0.2 and 0.4, respectively. When TP is equal to 0.8, BIC-seq2 ranks fifth. SPCNV ranks third at TPs of 0.2, 0.4 and 0.6, respectively. When TP is equal to 0.8, SPCNV ranks second. CNV-LOF detects the fewest losses at TP of 0.6 and 0.8, respectively. When TP is equal to 0.2 and 0.4, CNV-LOF ranks fourth. CNVnator ranks fifth at TPs of 0.2 and 0.4. When TP is sequentially equal to 0.6 and 0.8, CNVnator rank fourth and third, respectively. When TP is sequentially equal to 0.2 and 0.4, CNVkit detects the fewest losses, and ranks fifth and fourth at TPs of 0.6 and 0.8, respectively. On the whole, LDCNV detects more gains and losses than most comparison methods, which indicates that the performance of the proposed method is the most balanced in detecting gains and losses.

**FIGURE 7:**
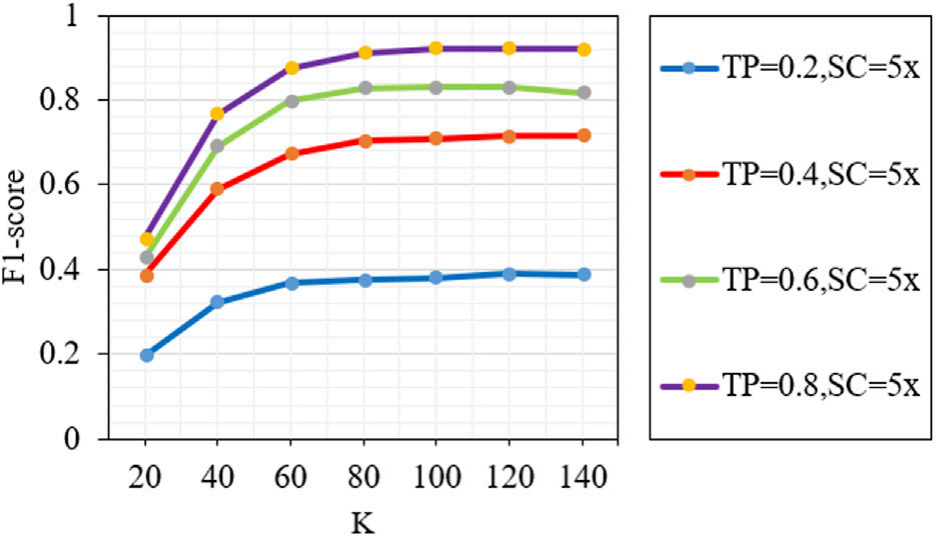
Analysis of the effect of different k parameters on the performance of the proposed method. TP and SC represent tumor purity and sequencing coverage, respectively.

The number of nearest neighbors (k) is a key parameter in the proposed method, which will affect the accuracy of detection results. Figure 7 describes the F1-scores of the proposed method under different k values (20, 40, 60, 80, 100, 120 and 140). We can see that the performance of the proposed method increases rapidly when the value of k is from 20 to 80, and the performance of the proposed method is basically stable when the value of k is from 100 to 140. The above analysis shows that if the value of k is greater than or equal to 100, the proposed method can achieve a good and stable performance.

### 3.2 Detection of real data from the 1,000 Genomes Project

**FIGURE 8:**
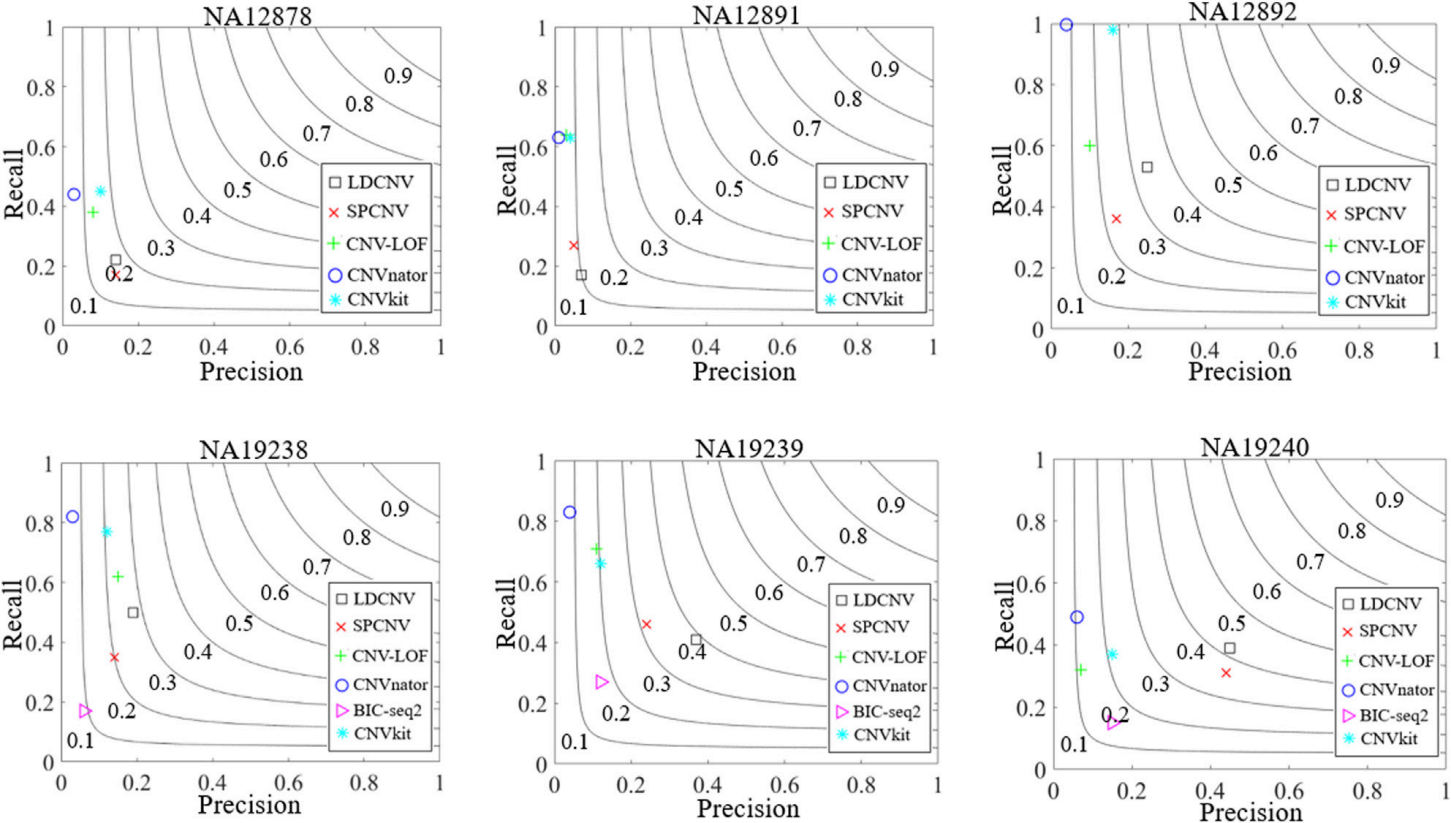
Comparison of the performance of LDCNV and five comparison methods in terms of recall, precision and F1-score on six real human data samples from 1000 Genomes Project. The F1-score levels are depicted with black arcs from 0.1 to 0.9.

To verify the performance of the proposed method on real data, six real human blood samples (NA12878, NA12891, NA12892, NA19238, NA19239, and NA19240) are selected from the 1000 Genomes Project (http://www.internationalgenome.org/). The DGV database records the partial detection results of these samples, which are regarded as ground truth to approximate the recall, precision, and F1-score of the six comparison methods. Figure 8 describes the detection results of each method in details. LDCNV obtains the best F1-score, precision and moderate recall in each sample. SPCNV’s F1-score ranks third in NA12878 and NA12892, and rank second in NA12891, NA19238, NA19239 and NA19240, respectively. CNV-LOF’s F1-score ranks fourth in NA12878, NA12891, NA12892 and NA19239, respectively. It ranks second and fifth in NA19238 and NA19240, respectively. F1-score of CNVnator rank fifth in NA12878, NA12891 and NA12892, and rank sixth in NA19238, NA19239 and NA19240, respectively. The F1-score of BIC-seq2 ranks fourth in NA19240, ranks fifth in NA19238 and NA19239, respectively. It does not detect the correct CNVs compared with the ground truth in the remaining three samples. CNVkit’s F1-scores rank second in NA12878 and NA12892, and rank third in NA12891, NA19238, NA19239 and NA19240, respectively. SPCNV’s precision ranks second five times, and ranks third in NA19238. CNV-LOF’s precision ranks fourth in NA12878, NA12891and NA12891, ranks second in NA19238, and ranks fifth in NA19239 and NA19240, respectively. CNVnator obtains the lowest precision in each sample, the reason of which has been analyzed in the simulation data experiment. In NA19238, NA19239, and NA19240, BIC-seq2 ranks fifth, fourth and fourth, respectively. BIC-seq2’s precision ranks third five times, and ranks fourth in NA19238. CNV-LOF, CNVnator and CNVkit obtain better recall than other comparison methods in most samples, but their precision is very low. SPCNV gets moderate recall, and BIC-seq2 gets lowest recall in each sample. The recall of each method is better than the precision in most samples. Overall, the performance of LDCNV is the most balanced between recall and precision.

### 3.3 Comparison of running time

The massive amount of NGS data brings great challenges to the execution efficiency of each method. To this end, the proposed method and other five comparison methods are tested on 50 samples, while they run on a PC with 2.50 GHz CPU and 16.0 GB RAM. Here, the running time is used to evaluate the execution efficiency of each method, and the running time of each method is calculated as the mean of 50 samples. The comparison results are recorded in Table 1. In terms of running time, LDCNV is the fastest in all methods, followed by SPCNV, CNVnator, CNV-LOF, CNVkit and BIC-seq2. In general, LDCNV is an efficient and reliable CNV detection method.

**TABLE 1:** Comparison of running time of six methods.

Method	LDCNV	SPCNV	CNV-LOF	CNVnator	BIC-seq2	CNVkit
Running time (s)	14.6	14.81	15.49	15.15	75.72	53.42

## 4 Discussion and conclusion

In the study of genomic structural variation detection, the detection of CNV is an important component for us to analyze genomic variation. In this work, a novel method called LDCNV is proposed for CNV detection using NGS data. LDCNV is developed based on the core idea of local distance, and utilizes a local idea to identify CNVs, which is different from conventional detection methods that use the idea of global statistical modeling to predict CNVs. LDCNV can effectively extract RD profiles from cancer samples, filter abnormal locations, calibrate GC content, remove noise, and transform the dimension of RD profiles. Based on the pretreated RD profile, the LDS is constructed by using the ratio of the mean distance between each RD and its nearest neighbor to the mean distance between the nearest neighbors, which fully reflects the degree of isolation between each RD and its k nearest neighbors. LDCNV fully considers the fluctuation range of the overall RDs and the difference between the local RDs, which is nonlinearly transformed into an LDS that can objectively reflect the degree of abnormality and is more conducive to detecting CNVs than directly using the RD. The LDS can objectively reflect the abnormal degree of each RD. At the same time, the LDSs are used to fit a normal distribution, the distribution function of which is used to evaluate the *p*-value of each LDS, and the hypothesis test is used to predict the CNVs.

Simulation and real data experiments are designed to verify the performance of the proposed method, and five methods of the same type are selected for comparison. In the simulation data experiment, LDCNV achieves the best balance between recall and precision, gets the best TPR and FDR in the detection of CNVs of different sizes, and obtains the best operation efficiency. Then, the selection of k parameters is further analyzed and discussed, which can help users obtain good performance in the process of using the software. In real data application scenarios, six real human blood samples are selected to test the performance of six methods. LDCNV achieves the best F1-score in each sample, which further reflects that the proposed method is an effective and reliable CNV detection tool.

While applying the proposed method, it is found LDCNV has some shortcomings that need to be improved in future work. The function of LDCNV needs to be further expanded. Its function currently only allows for the detection of a single sample, and does not support the detection of paired or multiple samples, which results in the inability to distinguish the heritability of CNVs or identify recurrent CNVs. LDCNV only extracts two features of RD, and the next step will incorporate other features, such as base alignment quality and GC content, to improve the detection accuracy. The resolution of the proposed method needs to be further improved, which can reduce the false positive positions in the detection results and improve the accuracy of identifying the boundary of CNVs. We will extract split reads and the insertion size of paired reads together with RDs to locate the breakpoint position and further improve the accuracy of CNVs. At the same time, we will introduce detection algorithms from other fields to improve the accuracy of the proposed method (Tuo et al., 2020). In future work, the above issue will be addressed, which enables LDCNV to be applied to numerous scenarios.

## Data Availability

The datasets presented in this study can be found in online repositories. The names of the repository/repositories and accession number(s) can be found in the article/Supplementary Material.
