# Supplementary material for: Detection of copy number variations based on a local distance using next-generation sequencing data
Source: Front Genet. 2023 Sep 22;14:1147761. doi: 10.3389/fgene.2023.1147761 (PMC10556732; doi:10.3389/fgene.2023.1147761)
Supplement: Supplementary file 1 [file DataSheet1.docx]

Supplementary Material

# Supplementary Figure 1. The relationship between RD, bins and breakpoints.


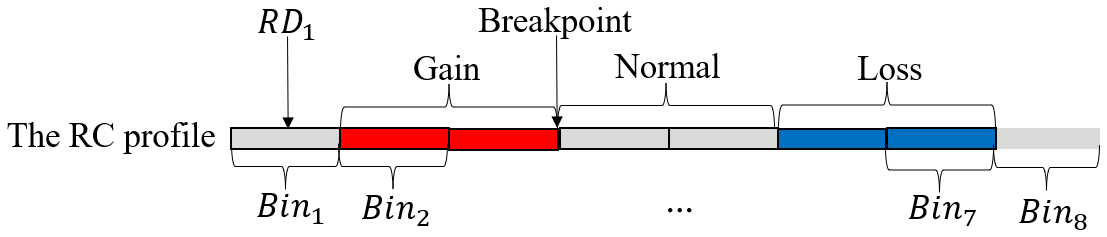


# Supplementary Figure 2. Obtain the screenshot of the software package ([LDCNV.tar.gz](https://github.com/gj-123/LDCNV/releases/download/v1.0/LDCNV.tar.gz)).


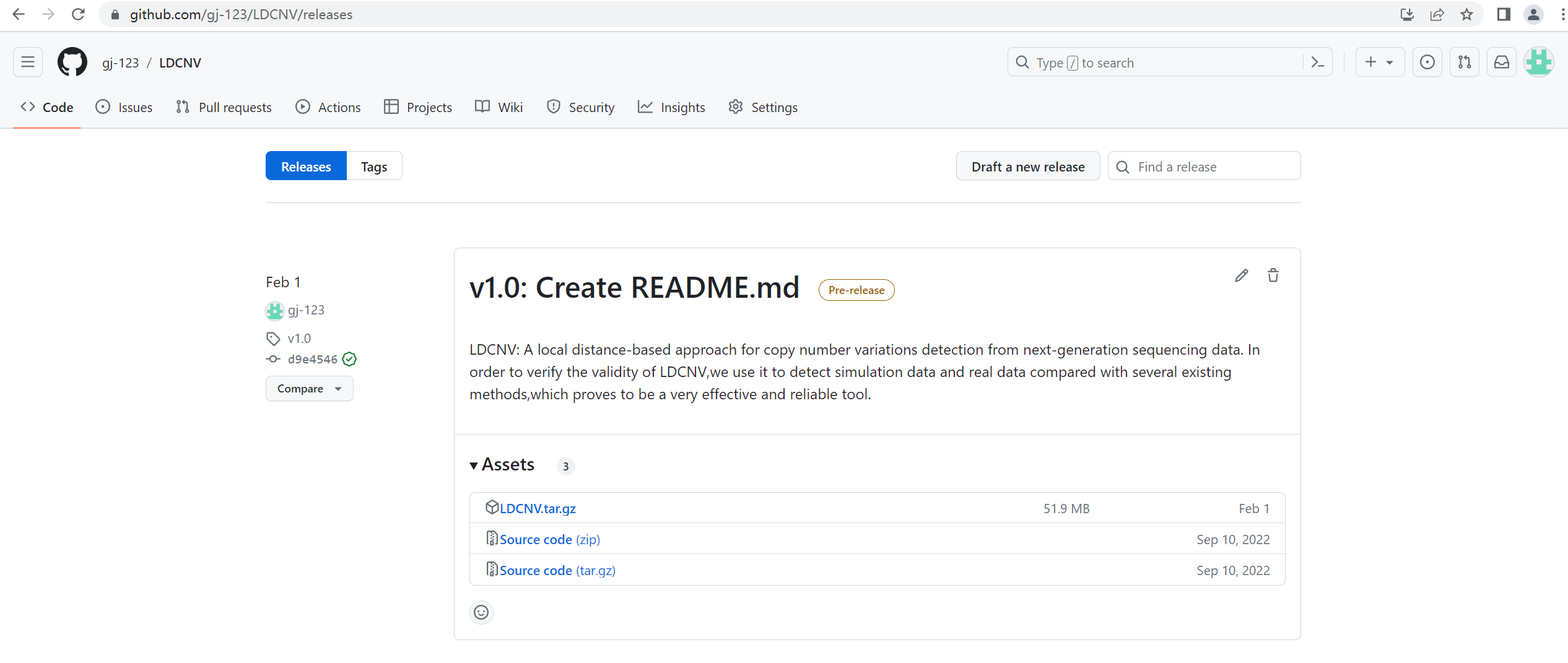


# Supplementary Table 1. Performance analysis of the proposed methods under different bin sizes.

| bin size | 1000 | 2000 | 3000 | 4000 | 5000 |
| --- | --- | --- | --- | --- | --- |
| Recall | 0.91 | 0.87 | 0.74 | 0.77 | 0.7 |
| Precision | 0.61 | 0.98 | 0.99 | 1 | 1 |
| F1-score | 0.73 | 0.92 | 0.85 | 0.87 | 0.82 |

# Supplementary Table 2. Comparison of four methods on two ovarian cancer samples.

| Sample | LDCNV | SPCNV | CNV-LOF | BIC-seq2 |
| --- | --- | --- | --- | --- |
| EGAR00001005450_1752_1 | 307 | 279 | 16 | 254 |
| EGAR00001005451_1752_2 | 1084 | 656 | 451 | 566 |

# Supplementary Table 3. Comparison of three methods on four sequencing coverage (30x, 40x, 60x and 100x) samples.

| Sample | Index | LDCNV | SPCNV | CNV-LOF |
| --- | --- | --- | --- | --- |
| 30x | recall | 0.71 | 0.79 | 0.5 |
|  | precision | 0.56 | 0.46 | 0.67 |
|  | F1-score | 0.63 | 0.58 | 0.62 |
| 40x | recall | 0.79 | 0.71 | 0.64 |
|  | precision | 0.73 | 0.56 | 0.9 |
|  | F1-score | 0.76 | 0.63 | 0.75 |
| 60x | recall | 0.71 | 1 | 0.79 |
|  | precision | 0.4 | 0.29 | 0.92 |
|  | F1-score | 0.51 | 0.45 | 0.85 |
| 100x | recall | 0.57 | 0.71 | 0.64 |
|  | precision | 0.12 | 0.08 | 0.9 |
|  | F1-score | 0.2 | 0.15 | 0.75 |
